# Worsened outcome in patients with pancreatic ductal carcinoma on long-term diabetes: association with E-cadherin1 (CDH1) promoter methylation

**DOI:** 10.1038/s41598-017-18438-z

**Published:** 2017-12-22

**Authors:** Takeshi Saito, Hiroki Mizukami, Satoko Umetsu, Chiaki Uchida, Wataru Inaba, Makoto Abe, Kazuhisa Takahashi, Kazuhiro Kudo, Chieko Itabashi, Soroku Yagihashi, Kenichi Hakamada

**Affiliations:** 1 Department of Pathology and Molecular Medicine, Hirosaki University Graduate School of Medicine, 5 Zaifu-cho, Hirosaki, 036-8562 Japan; 2 Department of Gastroenterological Surgery, Hirosaki University Graduate School of Medicine, 5 Zaifu-cho, Hirosaki, 036-8562 Japan

## Abstract

Prevalence of pancreatic ductal carcinoma (PDC) is nearly twice in patients with diabetes mellitus, but the reason for this close association remains obscure. Recently promoter methylation of E-cadherin1 (CDH1) and CDKN2A genes, encoding E-cadherin and P16 respectively, are invoked in development of PDC. It is still unclear whether diabetes affects such epigenetic changes and malignant behavior in PDC. In this study, we studied whether diabetes influences the clinico-pathological profile and methylation status of CDH1 and CDKN2A genes in patients with PDC. PDC subjects were divided into 3 groups; 59 cases without diabetes (non-DM), 17 cases with short-term diabetes (short-DM)(diabetes duration 3 yrs>) and 33 cases with long-term diabetes (long-DM)(≧3 yrs). Compared to non-DM or short-DM, long-DM was associated with a higher histological grade of malignancy and a higher tumor stage. Promoter methylation of both CDH1 and CDKN2A was encountered more frequently in PDC patients with long-DM than non-DM or short DM. Cases with CDH1 promoter methylation showed reduced E-cadherin expression and worsened survival. We consider that the presence of long-DM has a negative impact on the prognosis of PDC patients which may be relevant to a high frequency of promoter methylation of CDH1.

## Introduction

Pancreatic ductal carcinoma (PDC) is a highly aggressive tumor and its prevalence is increasing worldwide^1^. Surgical resection is an ultimate choice for the curable treatment, but it is limited to only 10–20% of cases^2^. Diabetes is long known to be a risk for the development of PDC. Huxley reported that type 2 diabetes was associated 1.2 times more frequently with PDC^3^, while Japanese study group reported that PDC was 1.8 times more frequent in patients with diabetes compared to non-diabetic subjects^4^. Despite such close connection between diabetes and PDC, there is not sufficient information on how diabetes is involved in the pathogenesis of PDC or how diabetes influences the prognosis or malignant behavior of the tumor in patients with PDC. While some investigators failed to detect a significant impact of diabetes on the prognosis of PDC patients, others found a shortened survival which correlated with the duration of type 2 diabetes^5–9^. In the latter study, worsened overall survival was demonstrated in patients with PDC complicated with diabetes for over 4 years^7,9^. However, no reason for the poor prognosis in PDC patients with diabetes was provided in those studies.

There is a growing evidence that the molecular alterations under both genetic and epigenetic basis are strongly related to the occurrence of PDC and its malignant behavior^10,11^. Environmental factors as well as the presence of diabetes may be possible to exert such molecular pathway^12–18^. During the last two decades, methylation specific polymerase chain reaction (MS-PCR) has well been applied to examine the role of epigenetic changes in the development and progression of various cancers^19–23^. With this method, methylation status of CpG islands of promoter region can be evaluated to determine whether the methylation elicits inactivation or silencing of the target genes. In fact, it was shown that promoter methylation of E-cadherin1 (CDH1) and cyclin-dependent kinase inhibitor 2A (CDKN2A) genes, encoding E-cadherin for an epithelial cell adhesion molecule and P16 for cell cycle regulator, respectively, correlated with the high occurrence of pancreatic cancer or rapid progression of the disease^24,25^. Although promoter methylation of CDKN2A and CDH1 was demonstrated in 14–39% and 7% of PDC cases, respectively^20,26^, it remains unclear whether concomitant presence of diabetes has any influences on the above epigenetic changes or invasive or metastatic natures of PDC.

In this study, we evaluated the methylation status of CDH1 and CDKN2A promoters in PDC tissues and non-neoplastic tissues obtained from patients with or without diabetes and explored to clarify whether diabetes influences tumor behavior and prognosis of the patients. The selection of these genes may be explained by following reasons. First, promoter methylation of CDH1 and CDKN2A are known to regulate the expressions of E-cadherin and P16 in PDC^20^. Secondly, frequency of promoter methylation is reported to be less than half in PDC enabling to evaluate the increase in diabetes if there is any^25,26^. Thirdly, reliable primer sets are available for formalin fixed paraffin embedded (FFPE) samples in MS-PCR assay^19^. Lastly, there is a high probability of promoter methylation of the above genes in diabetic state^27–29^, because promoter methylation is shown to occur in various tissues in patients with diabetes^15–18^.

## Results

### PDC in long-term diabetes is likely a high grade malignancy

Average age, gender ratio, body mass index, and the time from the diagnosis of PDC to surgical resection were comparable among the groups suffering from PDC (Table 1). Duration of diabetes was much longer in long-term diabetes (long-DM; 13.6 ± 1.2 years) than in short-term diabetes (short-DM; 1.8 ± 0.2 years) (p < 0.05). Duration of diabetes in long-DM was also comparable to that of type 2 diabetic subjects without PDC (T2DM). Elevated glycated hemoglobin levels prior to operation were significantly reduced at post-operation most prominently in short-DM, while the changes were only modest in long-DM (Table 1). There was no specific difference in the diabetes treatment among the diabetic groups.

**Table 1:** Clinical and pathological profiles of examined subjects.

	Cases with PDC			Cases without PDC	
	non-DM	short-DM	long-DM	Control	T2DM
Number (Male/Female)	59 (23/36)	17 (11/6)	33 (14/19)	23 (13/10)	19 (10/9)
Age (yrs)	66.3 ± 1.1	68.1 ± 2.1	68.8 ± 1.4	63.8 ± 2.0	67.1 ± 2.7
Body mass index	22.7 ± 0.5	22.3 ± 0.7	21.7 ± 0.5	22.0 ± 0.8	24.2 ± 1.9
Diabetes duration (yrs)		1.8 ± 0.2	13.6 ± 1.2^*^		12.4 ± 2.1^*^
HbA1c (NGSP, %) (pre-operation)	5.7 ± 0.1	8.9 ± 0.6^†^	8.1 ± 0.3^†^	5.3 ± 0.2	7.2 ± 0.4^†^
HbA1c (NGSP, %) (post-operation)	5.9 ± 1.0	6.5 ± 0.2^§^	7.5 ± 0.2^*,†^		
ΔHbA1c (%) (pre-operation – post-operation)	0.2 ± 0.1	−2.3 ± 0.6^#^	−0.6 ± 0.3^**^		
Diabetes therapy:					
Diet		11.8% (2/17)	6.1% (2/33)		6.7% (1/15)
OHA		64.7% (11/17)	87.9% (29/33)		46.7% (7/15)
Insulin		29.4% (5/17)	30.3% (10/33)		46.7% (7/15)
Tumor size (mm)	38.5 ± 2.4	35.9 ± 4.2	38.4 ± 2.6		
Histological grade:					
wel-mod	78.0% (46/59)	76.5% (13/17)	42.4% (14/33)		
por	22.0% (13/59)	23.6% (4/17)	57.6% (19/33)^†,††^		
T stage:					
T1-T2	11.9% (7/59)	5.9% (1/17)	0% (0/33)		
T3-T4	88.1% (52/59)	94.1% (16/17)	100% (33/33)^**,††^		
N stage:					
N0	33.9% (20/59)	52.9% (9/17)	30.3% (10/33)		
N1	66.1% (39/59)	47.1% (8/17)	69.7% (23/33)		
ly-factor	1.93 ± 0.11	1.86 ± 0.18	2.18 ± 0.13		
v-factor	1.94 ± 0.11	1.82 ± 0.19	2.30 ± 0.10^**,††^		
Recurrence by metastasis	55.9% (33/59)	41.2% (7/17)	78.8% (26/33)^**,††^		

NGSP; National Glycohemoglobin Standardization Program, NAC; neo-adjuvant chemotherapy, wel; well-differentiated adenocarcinoma, mod; moderate-differentiated adenocarcinoma, por; poorly-differentiated adenocarcinoma, OHA; oral hypoglycemic agent. Mean ± SE, *p < 0.01 vs short DM, ^†^p < 0.01 vs non-DM, ^§^p < 0.01 vs HbA1c (%) (pre-operation), ^#^p < 0.01 vs non-DM, p < 0.05 vs long-DM, **p < 0.05 vs non-DM, ^††^p < 0.05 vs short-DM.

Tumor size at the time of operation was all comparable among the groups (Table 1). There was an increased frequency of PDC cases with a higher histological grade of malignancy (por) in long-DM (57.5%) compared to short-DM (23.5%) or non-DM (22%) (p < 0.01 for both). Tumor stage based on TNM classification (UICC, 7^th^ Edition)^30^ was all T3-T4 in 33 cases of long-DM, whereas 12% and 6% cases remained T1-T2 stage in non-DM and short-DM, respectively. There was no difference in the frequency of lymph node metastasis among the groups. Although average score of ly-factor in PDC was higher in long-DM compared to non-DM and short-DM, the difference did not reach significant difference (p = 0.15 vs non-DM or short-DM). In contrast, PDC in long-DM showed a higher average score of v-factor compared to non-DM or short-DM (p < 0.05, vs non-DM or short-DM). In addition, recurrent distant organ metastasis in post-operation was more common in long-DM (78.8%) than that in short-DM (41.2%) or non-DM (55.9%) (p < 0.05 for both).

### Promoter methylation is accelerated in long-term diabetes

**Figure 1:**
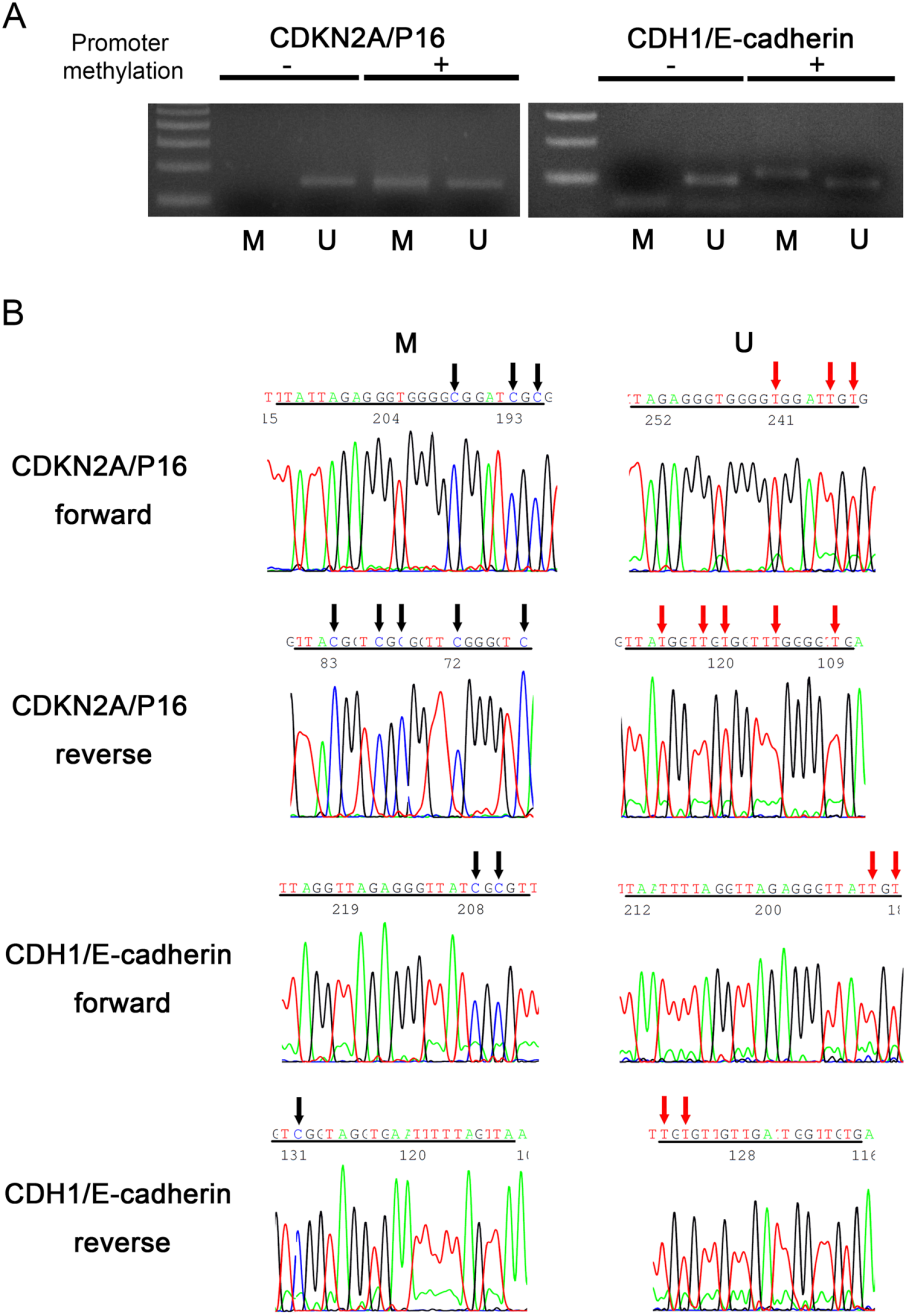
Analysis by methylation-specific PCR in PDC. The subjects bearing promoter methylation of CDKN2A and CDH1 showed both methylated and unmethylated bands (**A**). Non-methylated tumor exhibited only unmethylated band. U = unmethylated, M = methylated (See also full-length gels for each gene in Supplementary Figure S2). Sequencing of the amplified PCR-products indicated methylated base of cytosine as C (black arrows), whereas C in unmethylated DNA was converted to thymidine (T) after bisulfide treatment (red arrows) (**B**). Lines under nucleotide sequence indicate primer sequence.

MS-PCR distinguished methylated (M) and unmethylated (U) promoters by specific primers designed for CDKN2A and CDH1 (Fig. 1A). Positive bands for both M and U indicate the subjects for promoter methylation status. Promoter methylation was confirmed by DNA sequencing which identified the absence of conversion after bisulfide treatment from C to T at CpG site in the sequence of CDH1-U and CDKN2A-U promoter regions (Fig. 1B). The reliability of the conversion was further verified by direct DNA sequencing for CDH1 and CDKN2A in each 10 cases. Both positive and negative control templates were also applied. MS-PCR products positive for CDH1 showed methylation in all CpG islands (100%) at 2 CpG sites with forward and one site with reverse primer sequence. In CDKN2A, all 5 sites of CpG island completely methylated with reverse primer (100%), while all 10 cases showed positive at 3 sites of CpG islands with forward primer sequence (Supplemental Table S1). From these results, it is likely that the presence of methylation band in MS-PCR indicates the high frequency of CpG methylation of target genes.

Frequency of cases positive for CDKN2A promoter methylation in the PDC tissue was increased in long-DM at 30% (10/33 subjects) compared to 10% (6/59 subjects) in non-DM and 6% (1/17 subjects) in short-DM (p < 0.05 vs non-DM and short-DM) (Table 2). CDKN2A promoter methylation was not detected in non-neoplastic tissues adjacent to cancerous area, or tissues of subjects without PDC.

**Table 2:** Promoter methylation analysis.

	CDKN2A/P16	CDH1/E-cadherin
	Promoter methylation (+)	Promoter methylation (+)
**Cases with PDC**		
**non-DM**		
Tumor (n = 59)	10% (6/59)	22% (13/59)
Non-tumor (n = 59)	0% (0/59)	7% (4/59)
**short-DM**		
Tumor (n = 17)	6% (1/17)	18% (3/17)
Non-tumor (n = 17)	0% (0/17)	6% (1/17)
**long-DM**		
Tumor (n = 33)	30% (10/33)^*^	61% (20/33)^†^
Non-tumor (n = 33)	0% (0/33)	30% (10/33)^§^
**Cases without PDC**		
T2DM (n = 19)	0% (0/19)	37% (7/19)^#^
Control (n = 23)	0% (0/23)	0% (0/23)

*p < 0.05 vs non-DM (Tumor) and short-DM (Tumor), ^†^p < 0.01 vs non-DM (Tumor) and short-DM (Tumor), ^§^p < 0.01 vs non-DM (Non-tumor), p < 0.05 vs short-DM (Non-tumor), ^#^p < 0.01 vs Control

In contrast to CDKN2A, methylation of CDH1 promoter was more commonly encountered in PDC tissues in long-DM at a rate of 61% (20/33 subjects), 22% (13/59 subjects) in non-DM, and 18% (3/17 subjects) in short-DM in the order of frequency (p < 0.01 long-DM vs non-DM and short-DM). It is of note that non-neoplastic tissues or subjects without PDC also showed methylation in 30% (10/33 subjects) in long-DM, 7% (4/59 subjects) in non-DM, 6% (1/17 subjects) in short-DM and 37% (7/19 subjects) in T2DM. Promoter methylation was not detected in tissues obtained from PDC-free non-diabetic subjects (0/23 subjects).

### E-cadherin expression, but not P16, is reduced in long-term diabetes

**Figure 2:**
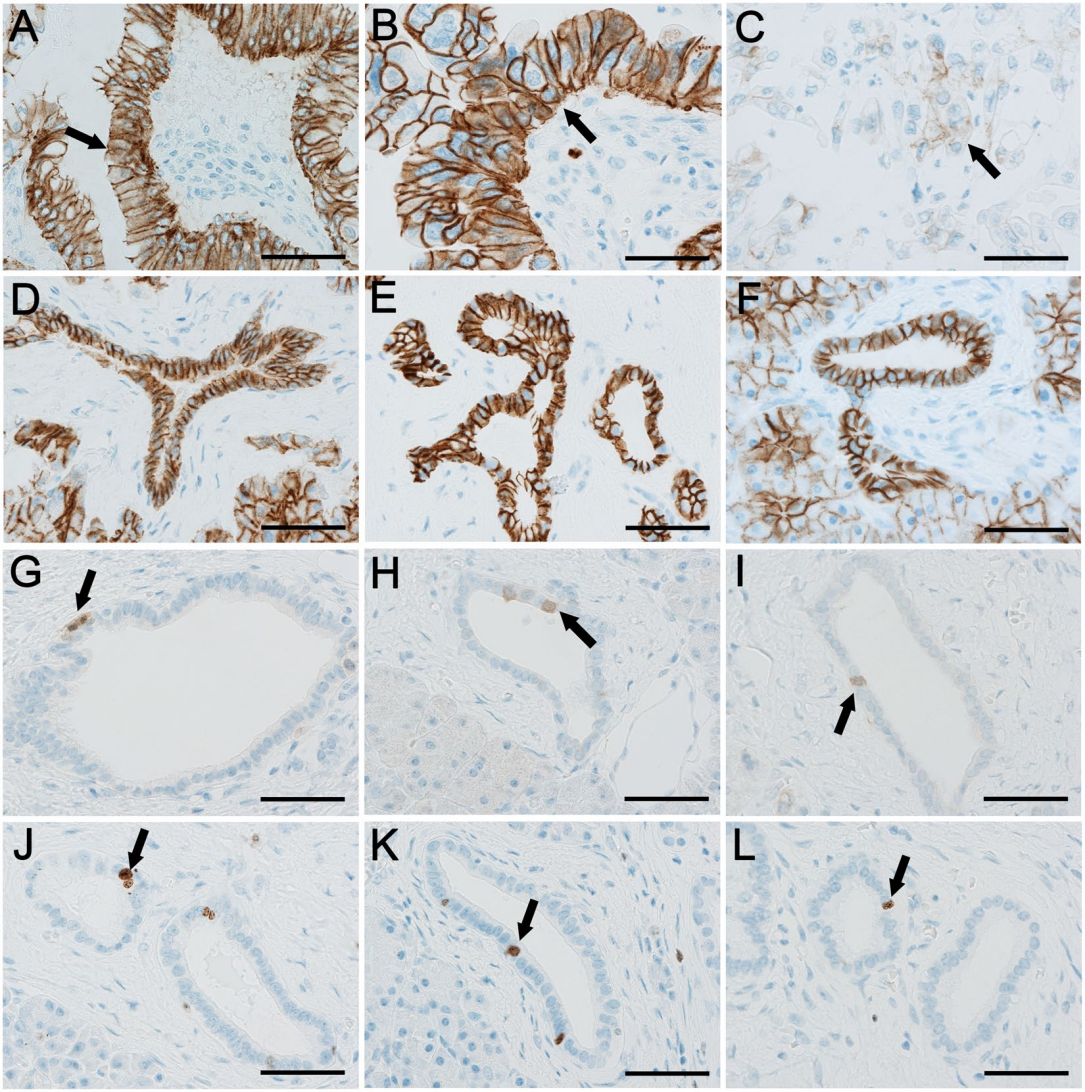
Expression of E-cadherin/CDH1 and P16/CDKN2A in PDC subjects. Diffuse membranous expression of E-cadherin/CDH1 was apparent in tumor cells (arrow) of non-DM (**A**) and short-DM (**B**) whereas the expression was equivocal (arrow) in long-DM showing diffuse invasive growth (**C**). E-cadherin/CDH1 was uniformly expressed in normal ductal structure adjacent to tumor tissues in non-DM (**D**), short-DM (**E**) and long-DM (**F**). The expression of P16/CDKN2A was sparse in the nuclei of tumor cells (arrow) in non-DM (**G**), short-DM (**H**) and long-DM (**I**) and there were no differences among groups. The expression in normal ducts (arrow) adjacent to PDC was comparable in non-DM (**G**), short-DM (**H**) and long-DM (**I**). Bar represents 50 μm in each.

**Figure 3:**
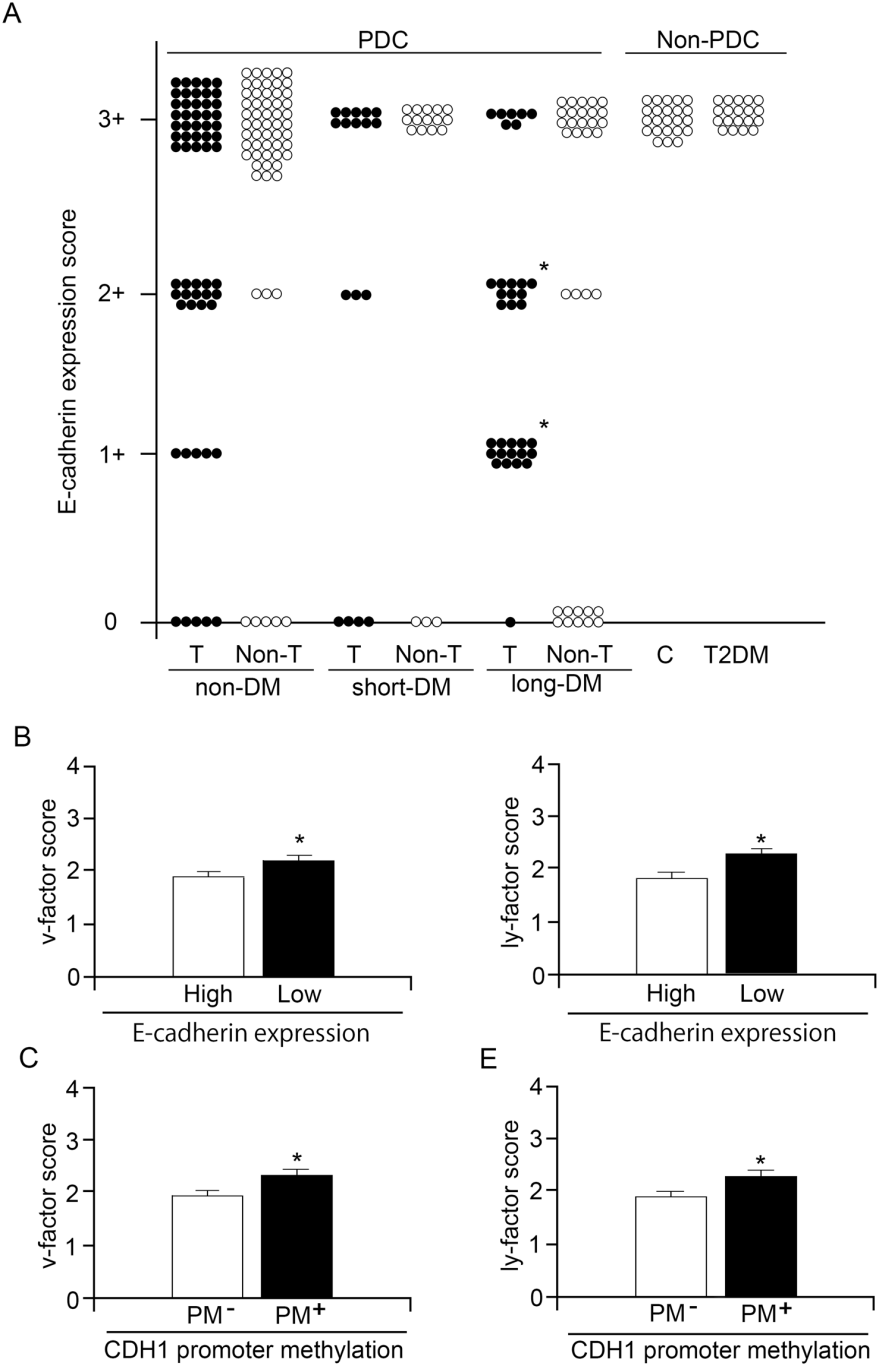
Scattered plots for E-cadherin/CDH1 expression in the investigated groups. Decreased membrane expression of E-cadherin/CDH1 (Score 2≧) was evident in PDC with long-DM (*p < 0.01 vs non-DM and short-DM) (**A**). The v- and ly-factor scores as markers of venular and lymph vessel invasion were significantly increased in a group with low E-cadherin expression (Score 2≧) compared to a group with high expression (Score 3) (*p < 0.05) (**B**). Similarly, group with positive CDH1 promoter methylation showed significant increases in v- and ly-factors compared to group with negative CDH1 promoter methylation (*p < 0.05) (**C**).

The histology of examined pancreas tissues stained with H&E is shown in Supplemental Fig. 1. There was aggressive growth of adenocarcinoma with irregular ductal structures mixed with dense fibrous stroma in all PDC lesions (Supplementary Figure 1A,B). In PDC with long-DM, there often appeared to be invasive growth of diffusely dispersed atypical cells with solid small nests of tumor cells (Supplementary Figure 1C). In adjacent non-neoplastic areas in each group, ducts were composed of mono-layered normal epithelial cells (Supplementary Figure 1D–F). Immunohistochemical staining revealed the expression of E-cadherin on the cell membrane of epithelial tumor cells, non-neoplastic ductal cells, islet cells and acinar cells (Fig. 2A–F). The intensity of E-cadherin expression in PDC appeared to be less in a group of long-DM compared to short-DM or non-DM. Semiquantitative evaluation of cancerous tissues disclosed reduced membrane expression of E-cadherin (score ≤ 2) notably in long-DM at 78% (25/32 subjects), and to lesser extent in non-DM (35%, 19/54 subjects) and short-DM (23%, 3/13 subjects) (Fig. 3A). Non-cancerous areas also showed a trend toward reduced expression of E-cadherin in long-DM compared to non-DM and short-DM which exhibited strongly positive reaction to E-cadherin. When we separated PDC groups into high E-cadherin expression group (score = 3, n = 52) and low expression group (score ≤ 2), (n = 47), there was a significant association between low expression of E-cadherin (≤2+) and the presence of CDH1 promoter methylation (p < 0.01). PDC subjects with negative CDH1 promoter methylation are likely to show high E-cadherin expression (67%, 44/66 cases) (See Supplemental Table S2). Furthermore, a group of low E-cadherin expression showed a significant increase in the scores of ly- and v-factor compared to a group of high E-cadherin expression (p < 0.05) (Fig. 3B). In a similar manner, CDH1-methylation-positive group (n = 36) showed significantly greater scores of ly- and v-factors compared to methylation-negative group (n = 73) (p < 0.05) (Fig. 3C).

P16 was expressed in the nuclei of tumour cells and non-neoplastic ductal cells, and also often faintly in the cytoplasm. There was no difference in the expression of P16 among all the groups either in tissues of PDC or adjacent non-PDC (Fig. 2G–L).

### Long-term diabetes is associated with short overall or disease-free survivals in pancreatic cancer

Univariate analysis for overall survival and disease-free survival showed that the tumour location in the head, lymph nodes metastasis, long-DM and methylation of CHD1 promoter were a significant risk for shorter survival (Table 3 and Supplemental Table S3). Age, gender, BMI, tumor size, tumor stage, glycated hemoglobin levels and CDKN2A promoter methylation did not affect the outcome. Multivariate analysis further confirmed that location in the pancreas head and presence of long-DM and CDH1 promoter methylation still contributed to worsened survival (Table 4 and Supplementary Table S4).

**Table 3:** Univariate analysis (Overall Survival).

Factor	median OS (month)	p-value
Age: ≤69 vs >69	25.0 vs 38.1	0.287
Male vs Female	26.5 vs 23.2	0.089
Location: Body-Tail vs Head	58.7 vs 19.6	0.002
BMI: <25 vs ≥25	25.0 vs 38.1	0.505
NAC: (−) vs (+)	26.5 vs 23.4	0.795
Adjuvant chemotherapy: (−) vs (+)	25.0 vs 23.4	0.593
Tumor size (mm): ≤40 vs >40	27.4 vs 17.7	0.100
T1-T2 vs T3-T4	24.7 vs 24.1	0.126
N: (−) vs (+)	38.7 vs 19.6	0.030
HbA1c (%): ≤7.0 vs >7.0	25.4 vs 25.0	0.801
Blood glucose (mmol/L): ≤11.1 vs >11.1	25.4 vs 25.0	0.709
Diet: (−) vs (+)	25.4 vs 17.9	0.562
OHA: (−) vs (+)	25.4 vs 25.0	0.642
Insulin: (−) vs (+)	25.4 vs 19.6	0.670
T2DM: (−) vs (+)	26.6 vs 24.1	0.673
long-DM: (−) vs (+)	28.7 vs 17.6	0.007
CDKN2A promoter methylation: (−) vs (+)	26.6 vs 22.1	0.368
CDH1 promoter methylation: (−) vs (+)	28.7 vs 17.1	0.002

OS; overall survival, BMI; body mass index, NAC; neoadjuvant chemotherapy, OHA; oral hypoglycemic agent.

**Table 4:** Multivariate analysis (Overall Survival).

Factor	Hazard ratio	95%CI	p-value
Location: Body-Tail vs Head	1.94	1.10–3.42	0.022
N: (−) vs (+)	1.63	0.94–2.84	0.082
long-DM: (−) vs (+)	1.71	1.01–2.90	0.045
CDH1 promoter methylation: (−) vs (+)	1.80	1.06–3.05	0.029

95% CI, 95% confidence interval.

**Figure 4:**
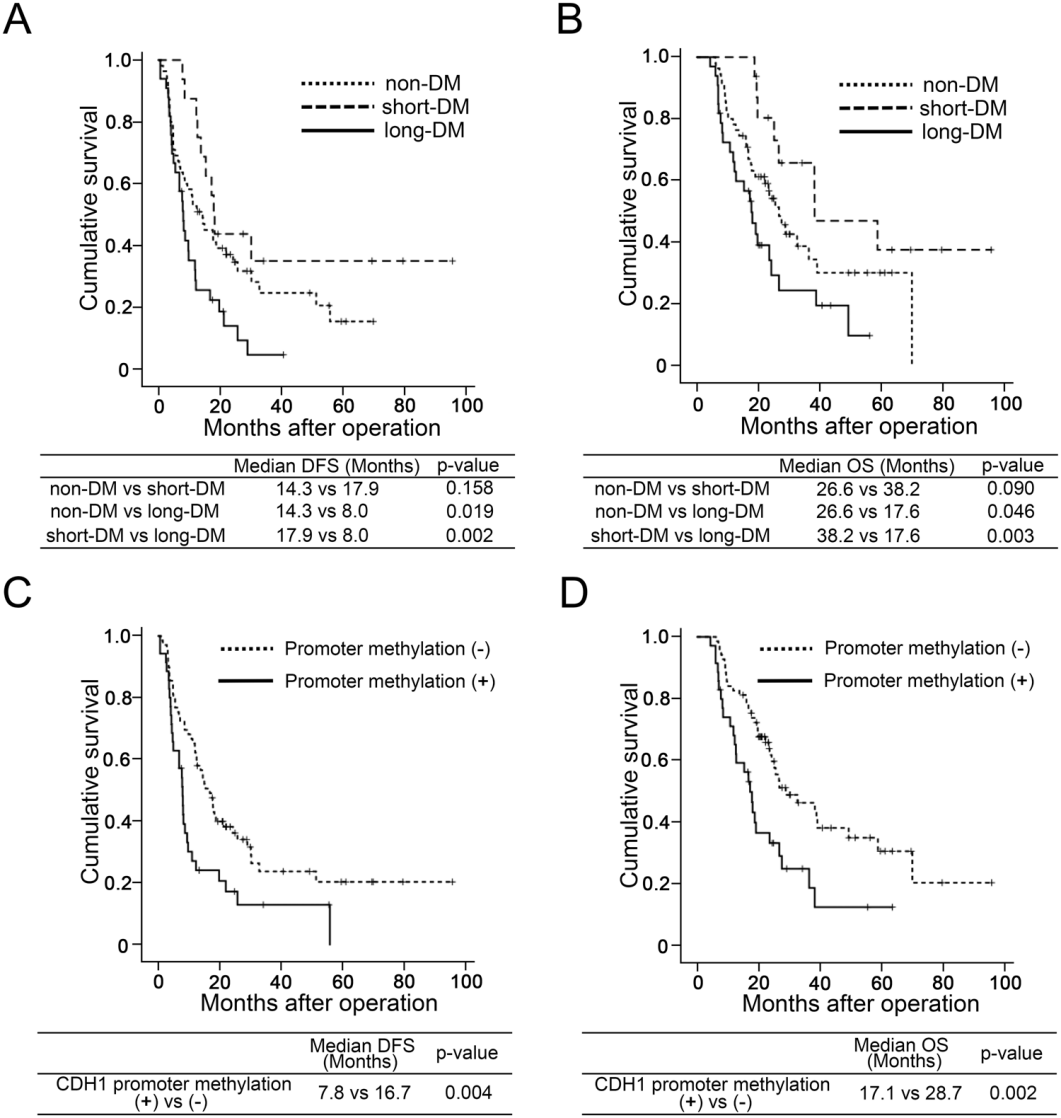
Survival curves based on disease free survival (DFS) and overall survival (OS). Survival rate of long-DM (solid line) was significantly low compared to non-DM (fine break line) and short-DM (rough break line) in both DFS (**A**) and OS (**B**). Group with CDH1 promoter showed methylation significantly worse DFS (**C**) and OS (**D**) in PDC subjects compared to methylation negative group.

Kaplan-Meier survival curve clearly indicated a shortened disease-free survival in long-DM compared to short-DM and non-DM (Fig. 4). Overall survival was also shortened in long-DM compared to non-DM and short-DM. Presence of CDH1 promoter methylation also shortened disease-free survival and overall survival (Fig. 4).

## Discussion

In this study, we first found that PDC commonly underwent methylation of both CDH1 and CDKN2A promoter genes, and positive cases for CDH1 and CDKN2A were more prevalent in long-DM compared to non-DM and short-DM. Direct sequencing of PCR products reproducibly confirmed the specific presence of methylation in CpG islands of the above genes. PDC subjects with positive CDH1 promoter methylation showed significantly greater scores of ly- and v-factors compared to those in methylation-negative subjects. Furthermore, promoter methylation of CDH1 well correlated with suppressed expression of E-cadherin but this was not the case of CDKN2A for P16. While E-cadherin expression may thus mainly be regulated by CDH1 gene, P16 expression appeared to be largely independent on promoter methylation of CDKN2A gene. It was thus suggested that protein expression was differently regulated between E-cadherin and P16. It is of a particular note that frequency of methylation-positive cases for CDH1 was more common in PDC with long-DM than previously reported data in general PDC groups in which the presence of diabetes was not specified^25,26^. Furthermore, we detected the methylation in 30% (10/33 cases) of non-neoplastic tissues in PDC patients with long-DM. It is thus likely that diabetes itself has a strong potential to elicit promoter methylation. We believe that this may not be due to contamination of sections containing tumor tissues during DNA extraction because we confirmed the absence of neoplastic cells on the first and the last sections of the samples.

We found that PDC patients with long-DM suffered from shortened disease-free survival and overall survival compared to patients without diabetes or with short-DM. Although direct connection of methylation with clinical outcome of PDC patients may be too speculative, lowered expression of cohesive protein of E-cadherin related to promoter methylation may possibly accelerate the detachment of tumor cells and promote vascular and lymph vessel invasion, resulting in distant metastasis and finally premature death in patients. Indeed, CDH1 promoter methylation-positive group in PDC patients with reduced E-cadherin expression showed greater values of ly- and v-factors compared to methylation-negative cases. Our uni- and multivariate analyses are also in keeping with such assumption, showing a high histological grade of malignancy and greater values of ly- and v-factors as well as frequent recurrences in PDC with long-DM.

Since only a single PDC case with short-DM (1/17 cases, 6%) showed promoter methylation of CDH1 or none for CDKN2A in non-neoplastic tissues, it is likely that the presence of long-term diabetes may be a major trigger for the epigenetic alterations of tumor suppressor genes. In this study the question why long-term diabetes is associated with high frequency of promoter methylation is not still answered. Triggers of promoter methylation are suggested to be aging, foods, smoking, exposure to chemical products, interferon or inflammation^12–14^. Continuous stimulation of the above factors may elicit the DNA modification. In diabetes, chronic production of pro-inflammatory cytokines, formation of advanced glycated end-products, and activation of inflammatory cells as well as other triggers may augment the production of oxidative stress^31–33^. Accumulation of excessive oxidative stress may in turn damage DNA including promoter regions, not any more repaired. Alternatively, chronic oxidative stress may enhance the activity of DNA methyltransferase, responsible for DNA promoter methylation^34^. Although our preliminary study on the markers for oxidative stress-related DNA damage cannot consistently underscore such hypothesis, future investigations on the expression of 8-hydroxy-2′-deoxyguanosine, γH2X or thioredoxin or thioredoxin-interacting protein will be warranted to disclose whether oxidative stress is involved in DNA methylation.

P16 has a pivotal role in the suppression of cell cycle progression^35^. It is known that CDKN2A promoter methylation occurs commonly in PDC, but its frequency in cancerous area was not consistent among PDC groups. In contrast to CDH1, cases positive for methylation of CDKN2A promoter in tumor tissues were much less even in long-DM (30%) and in non-DM (10%). Those changes were not paralleled with changes of P16 protein expression by immunohistochemistry which was comparable among all the groups. Therefore, although the presence of diabetes associates with high frequency of promoter methylation, promoter methylation of P16 may not be much involved in the malignant behavior of PDC and overall survival of PDC patients, different from E-cadherin. Our multivariate analysis could not find a significant impact of CDKN2A promoter methylation on the disease-free survival or overall survival.

Both overall survival and disease-free survival were significantly shortened in patients with long-DM. In contrast to long-DM, however, presence of short-DM yields paradoxical influences, showing a trend for improvement of survival in this group. Pannala *et al*. reported that glycated hemoglobin values were improved in PDC patients with short-DM subjects after the resection of the tumor by pancreato-duodenectomy, while none of the patients with long-DM showed improvement of diabetes^36^. Similar to the above report, our study showed that the glycated hemoglobin values were much improved in cases with short-DM (−2.3% HbA1c in average) (6/11 cases showed lower values than 6.5% of HbA1c), but not much so in patients with long-DM (only −0.6% HbA1c in average) after removal of the tumors. These findings suggest that diabetes may be secondary to PDC in short-DM^37^.

Development of PDC may be influenced by diabetes treatment. Previous studies showed that metformin treatment had suppressive effects on the tumors, whereas insulin or incretin based therapy may promote cell proliferation of PDC^38,39^. In this study, we could not find clear differences in the clinical behavior or pathological characteristics or methylation status among groups with different diabetes treatment. Multivariate analysis did not show that the type of diabetes treatment was an independent factor for disease-free survival or overall survival. Since the number of subjects in each group with specified treatment such as metformin was still small, further studies may be required to confirm the current results.

Apparently, there are several limitations in this study. First, only FFPE specimens were used in this study. Since alteration of DNA during tissue processing is known to occur, the results may be confounded by technical artifactual effects^40,41^. Although the quality of samples was confirmed by DNA sequencing and immunostaining for their reproducibility in this study, confirmation of the data using fresh samples may be warranted by future investigations. Another drawback in this study may be that the prevalence of PDC complicated with diabetes were not directly evaluated because of retrospective nature of the data. Future prospective study will be necessary to confirm our data. Our analysis was also limited to CDH1 and CDKN2A and it is impossible to speculate the whole scenario for the implication of promoter methylation only by these two genes in the development of PDC. Nevertheless, we believe that our study has provided important information on the close association of long-term diabetes and the promoter methylation of CDH1 with unfavorable prognosis. It is hoped that future studies will explore the possibility to develop effective demethylating agents for this disastrous disorder.

## Material and Methods

We recruited 109 patients with PDC who underwent pancreas resection and 42 age-matched autopsy patients free from PDC whose pancreas were available for the pathological and methylation studies from the archive files from 2006 to 2014 in Hirosaki University Hospital. Diabetic patients had a history of hyperglycemia that fulfilled the criteria of diabetes proposed by the Japan Diabetes Society^42^. Diagnosis of type 2 diabetes in a diabetic group was confirmed by the clinical record of the patients. Cases were excluded for investigations if they had been exposed to chronic glucocorticoid treatment, or pancreatic tissues had undergone autolysis or showed evidence of acute pancreatitis. Cases with a history of gastrectomy or under long-term treatment with anti-schizophrenic drugs were also excluded from the evaluation. 59 PDC cases were divided into 3 groups of non-diabetic PDC subjects (non-DM), 17 PDC cases with short-term diabetes (short-DM)(duration of diabetes < 3 yrs) and 33 PDC cases with long-term diabetes (long-DM)(duration ≧3 yrs). For comparison, 42 PDC-free autopsy patients were divided into 23 non-diabetic patients (Control) and 19 diabetic patients (T2DM). Type 2 diabetes was defined by the criteria of “type 2 diabetes”. Separation of diabetes into short- and long-term by 3 years was based on the previous report^9^. Clinicopathological parameters were compared from medical records of patients in Hirosaki University Hospital. Duration of PDC was defined from definite diagnosis of PDC to the date of the resection. Differences in the value of pre-operative HbA1c (%) (measured at the nearest to the operation) and post-operation HbA1c (%) (measured 3 month after the operation date) were defined as ΔHbA1c (%). All investigations and experiments were performed under the permission of the ethical committee of Hirosaki University Graduate School of Medicine (approved number #2016-0084) and were performed according to the guidelines of the Ethics Committee on human research samples at the Japanese Society of Pathology. Informed consent was obtained from all participants and/or their legal guardians.

### Histopathological assessment

Screening of pathological findings was performed with H&E sections in each subject. The pathological diagnosis of PDC was re-evaluated according to 2010 WHO classification of tumors of the digestive system and graded based on UICC TNM classification of malignant tumor (7^th^ edition) by three pathologists (H.M., K.K. and C.I.)^30^. Histological grade was divided into 3 categories of well differentiated carcinoma (wel), moderately differentiated adenocarcinoma (mod), and poorly differentiated adenocarcinoma (por), based on the degrees of tubular formation, mucin production and mitoses^30^. The highest grade in the sections represented the histological grade of the individuals regardless of the proportion^30^. Venous invasion was assessed on the tumor sections stained with Elastica-von Gieson. Lymphatic invasion was evaluated on the immunostained sections for podplanin. The degree of invasion to venules and lymph vessels was graded as 0 (none), 1 (0–3 sites), 2 (3–6 sites) and 3 (6 sites<) within 10 high power field.

### Genetic analysis

MS-PCR was carried out following the previous protocol^19^. Tumor area without hemorrhage, necrosis or serious inflammation and area adjacent to the tumor (6 cm^2^ in average) was selected for the evaluation. DNA was extracted from FFPE tissue sections (10 μm) following the protocol of DNA extraction kit for FFPE (Qiagen K.K., Tokyo, Japan). Subsequently bisulfite modification was conducted on the extracted samples with a commercially available kit (EpiTect Fast Bisulfite Conversion Kits, Qiagen K.K.), which were then subjected to MS-PCR reaction with identical primer sequence to Herman *et al*. using a Taq DNA polymerase designed for bisulfite PCR (EpiTaq™ HS, TAKARA BIO INC., Shiga, Japan)^37^. The amplicon was analyzed in electrophoresis with 3% agarose gel.

For the confirmation of methylation status of CDH1 and CDKN2A, MS-PCR products were ligated to the vector with a TOPO cloning kit (Thermo Fisher Scientific K.K., Yokohama, Japan). After blue-white selection, purified vector was digested with *Eco*RI. An about 150-bp insert clone was loaded onto a 3% agarose gel. Direct sequencing was carried out with ABI Prism 310 sequence analyser (Thermo Fisher Scientific K.K.) on the positive clone labelled with VIC dye sequence kit (Thermo Fisher Scientific K.K.).

### Immunohistochemical analysis

For immunohistochemistry, standard streptavidin–biotin technique was applied to the sections using Benchmark Ultra Automated Slide Preparation system (Ventana Medical Systems, Inc., Tucson, AZ, USA). The antibodies for P16 (Clone E6H4, prediluted, Ventana Medical Systems, Inc.), E-cadherin (clone NCH-38, 1:100 dilution, Agilent Technologies, Santa Clara, CA, USA) and podplanin (Clone D2-40, 1:4, Nichirei bioscience Inc., Tokyo, Japan) were used. Negative control stains were performed by omitting the primary antibodies or substituting nonimmune rabbit or swine sera. The number of cells showing clearly positive nuclear staining for P16 was counted in the areas with hot spot and recorded as the number of positive cells per all tumour cells in x40 power field. The positive reaction of E-cadherin was determined by the guideline of American Society of Clinical Oncology (ASCO)/College of American Pathologists (CAP) for human epidermal growth factor receptor 2 testing in breast cancer with slight modifications (Score 0: no staining, 1+: weak and incomplete membrane staining in less than 10% of the invasive tumor cells, Score 2+: weak to moderate and complete staining of the membrane in more than 10% or strong complete homogenous membrane staining in no more than 10% of invasive cancer cells, Score 3+: strong complete homogenous membrane staining in more than 10% of the invasive tumor cells)^43^. Less than score 2+ was judged as low expression of E-cadherin. Absolute lack of positive reaction in the whole section including surrounding the adjacent parenchyma was judged as “no expression”.

### Statistical analysis

All statistical analyses were conducted using SPSS software (version 24; IBM SPSS Inc., Chicago, IL, USA). Data were expressed as the mean with standard error (SE). Disease-free survival was defined as the time elapsed between surgical resection and tumor recurrence. Overall survival was calculated as the time between surgery and death from any cause. Continuous variables were compared with the Student’s *t* test or the Mann-Whitney *U* test. Categorical variables were compared by chi-square analysis or Mann-Whitney *U* test, where appropriate. Comparisons of average values between two groups were analysed by non-parametric Mann-Whitney *U* test. For multiple comparisons, the *Z* test with Bonferroni adjustment was used. Survival curves were calculated with Kaplan–Meier analysis, and *p* values were determined by the log rank test for censored survival data. Multivariate survival analysis was performed by the Cox proportional hazard model. All tests were two-tailed, and p value of <0.05 was considered statistically significant.

### Data Availability

The datasets generated during the current study are available from the corresponding author on reasonable request.

## Electronic supplementary material

Supplementary Information
