# Supplementary material for: Worsened outcome in patients with pancreatic ductal carcinoma on long-term diabetes: association with E-cadherin1 (CDH1) promoter methylation
Source: Sci Rep. 2017 Dec 22;7:18056. doi: 10.1038/s41598-017-18438-z (PMC5741711; doi:10.1038/s41598-017-18438-z)
Supplement: Supplementary file 1 — Supplementary Information [file 41598_2017_18438_MOESM1_ESM.doc]

Original Article for Scientific Reports

*Worsened outcome in patients with pancreatic ductal carcinoma on long-term diabetes: association with E-cadherin1 (CDH1) promoter methylation*

Takeshi Saito^1,2^, Hiroki Mizukami^1^, Satoko Umetsu^1,2^, Chiaki Uchida^1,2^, Wataru Inaba^1^, Makoto Abe^1^, Kazuhisa Takahashi^1^, Kazuhiro Kudoh^1^, Chieko Itabashi^1^, Soroku Yagihashi^1^, Kenichi Hakamada^2^

^1^Department of Pathology and Molecular Medicine, ^2^Department of Gastroenterological Surgery, Hirosaki University Graduate School of Medicine,

Address correspondence: Dr. Hiroki Mizukami

Department of Pathology and Molecular Medicine,

Hirosaki University Graduate School of Medicine,

5 Zaifu-cho, Hirosaki. 036-8562 Japan

Phone: 81-172-39-5025

Fax: 81-172-39-5026

E-mail; hirokim@hirosaki-u.ac.jp

Running Title; epigenesis of pancreatic carcinoma in diabetes

Main text: 3850 words

Disclosure: There is no conflict of interest in any of the listed authors

This study was supported in part by KAKENHI (Grants-in-Aid for Scientific Research) from the Japanese Ministry of Education, Culture, Sports, Science and Technology to HM (#15K09374) and Pancreas Research Foundation of Japan to HM.

**List of supplementary material online information**

**Supplementary Table S1.** Detection of methylated or unmethylated sites in specifically designed primers

**Supplementary Table S2.** Correlation between promoter methylation and protein expression of E-cadherin/CDH1 and P16/CDKN2A

**Supplementary Table S3.** Univariate analysis (Disease Free Survival)

**Supplementary Table S4.** Multivariate analysis (Disease Free Survival)

**Supplementary Figure S1.** Histology of examined subjects in H&E sections

**Supplementary Figure S2.** Original full-length gels of methylation specific PCR for CDKN2A and CDH1

**Supplementary Table S1. Detection of methylated or unmethylated sites in specifically designed primers**

| **Primer for methylated** | **CpG site** | **Number of methylated CpG sites (%, cases)** | | | | | |
| --- | --- | --- | --- | --- | --- | --- | --- |
| **promoter** |  | **5** | **4** | **3** | **2** | **1** | **0** |
| **CDKN2A/P16 Forward** | 3 | **-** | **-** | 100 (10/10) | 0 (0/10) | 0 (0/10) | 0 (0/10) |
| **CDKN2A/P16 Reverse** | 5 | 100 (10/10) | 0 (0/10) | 0 (0/10) | 0 (0/10) | 0 (0/10) | 0 (0/10) |
| **CDH1/E-cadherin Forward** | 2 | **-** | **-** | **-** | 100 (10/10) | 0 (0/10) | 0 (0/10) |
| **CDH1/E-cadherin Reverse** | 1 | **-** | **-** | **-** | **-** | 100 (10/10) | 0 (0/10) |

|  | **E-cadherin/CDH1** | |
| --- | --- | --- |
|  | **Promoter methylation (+) (n=33)** | **Promoter methylation (-) (n=66)** |
| **Low expression (≦2+)** | 75.6% (25/33) | 33.3% (22/66) |
| **High expression (3+)** | 24.2% (8/33) | 66.7% ^*^(44/66) |

**Supplementary Table S2. Correlation between promoter methylation and protein expression of E-cadherin/CDH1 and P16/CDKN2A**

*p<0.01, χ squared test.

|  | **P16/CDKN2A** | |
| --- | --- | --- |
|  | **Promoter methylation (+)** | **Promoter methylation (-)** |
| **P16/CDKN2A index** | 6.1±2.2 | 6.9±1.3 |

Supplementary Table S3. Univariate analysis (Disease Free Survival)

| **Factor** | **median DFS (month)** | **p-value** |
| --- | --- | --- |
| Age: ≦69 vs >70 | 12.1 vs 12.2 | 0.296 |
| Male vs Female | 13.7 vs 11.1 | 0.232 |
| Location: Body-Tail vs Head | 15.3 vs 10.7 | 0.036 |
| BMI: <25 vs ≧25 | 11.9 vs 14.3 | 0.401 |
| NAC: (-) vs (+) | 11.8 vs 18.2 | 0.282 |
| Adjuvant chemotherapy: (-) vs (+) | 12.6 vs 9.7 | 0.929 |
| Tumor size (mm): ≦40 vs >40 | 17.2 vs 7.8 | 0.002 |
| T1-T2 vs T3-T4 | 21.9 vs 11.8 | 0.153 |
| N: (-) vs (+) | 13.7 vs 7.8 | 0.031 |
| HbA1c (%): ≦7.0 vs >7.0 | 12.4 vs 9.6 | 0.654 |
| Blood glucose (mg / dl): ≦200 vs >200 | 12.4 vs 12.1 | 0.554 |
| Diet: (-) vs (+) | 12.1 vs 9.7 | 0.803 |
| OHA: (-) vs (+) | 12.4 vs 11.8 | 0.322 |
| Insulin: (-) vs (+) | 12.1 vs 9.6 | 0.200 |
| T2DM: (-) vs (+) | 14.3 vs 11.9 | 0.438 |
| long DM: (-) vs (+) | 15.3 vs 8.0 | 0.002 |
| CDKN2A promoter methylation: (-) vs (+) | 13.7 vs 5.4 | 0.236 |
| CDH1 promoter methylation: (-) vs (+) | 16.7 vs 7.8 | 0.004 |

DFS, disease free survival; BMI, body mass index; NAC, neoadjuvant chemotherapy; OHA, oral hypoglycemic agent.

Supplemental Table S4. Multivariate analysis (Disease Free Survival)

| **Factor** | **Hazard ratio** | **95%CI** | **p-value** |
| --- | --- | --- | --- |
| Location: Body-Tail vs Head | 1.66 | 1.02-2.68 | 0.041 |
| Tumor size (mm): ≦40 vs >40 | 2.33 | 1.44-3.75 | 0.001 |
| N: (-) vs (+) | 1.11 | 0.68-1.83 | 0.672 |
| long DM: (-) vs (+) | 1.97 | 1.21-3.22 | 0.007 |
| CDH1 promoter methylation: (-) vs (+) | 1.75 | 1.08-2.83 | 0.024 |

95% CI, 95% confidence interval.


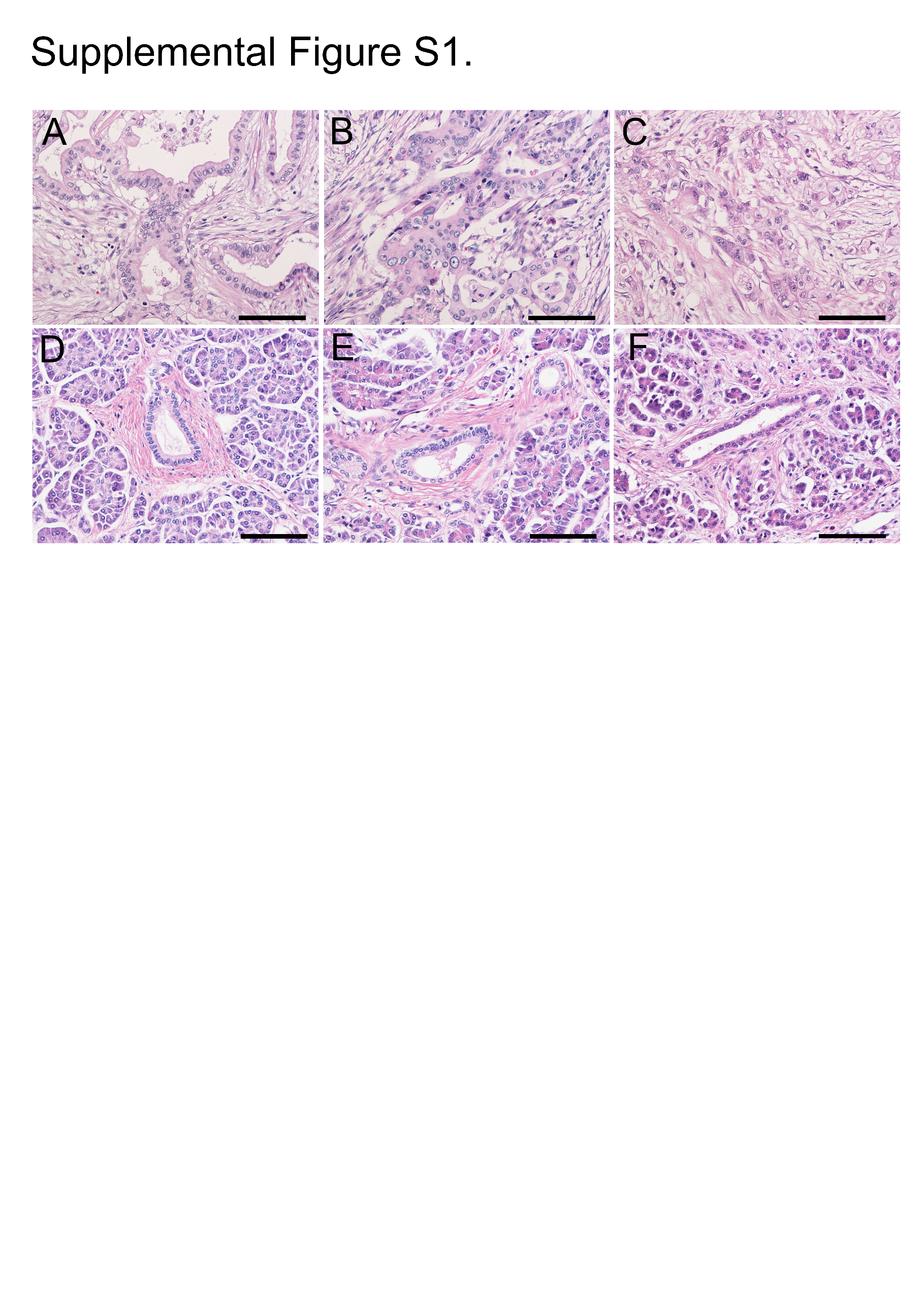


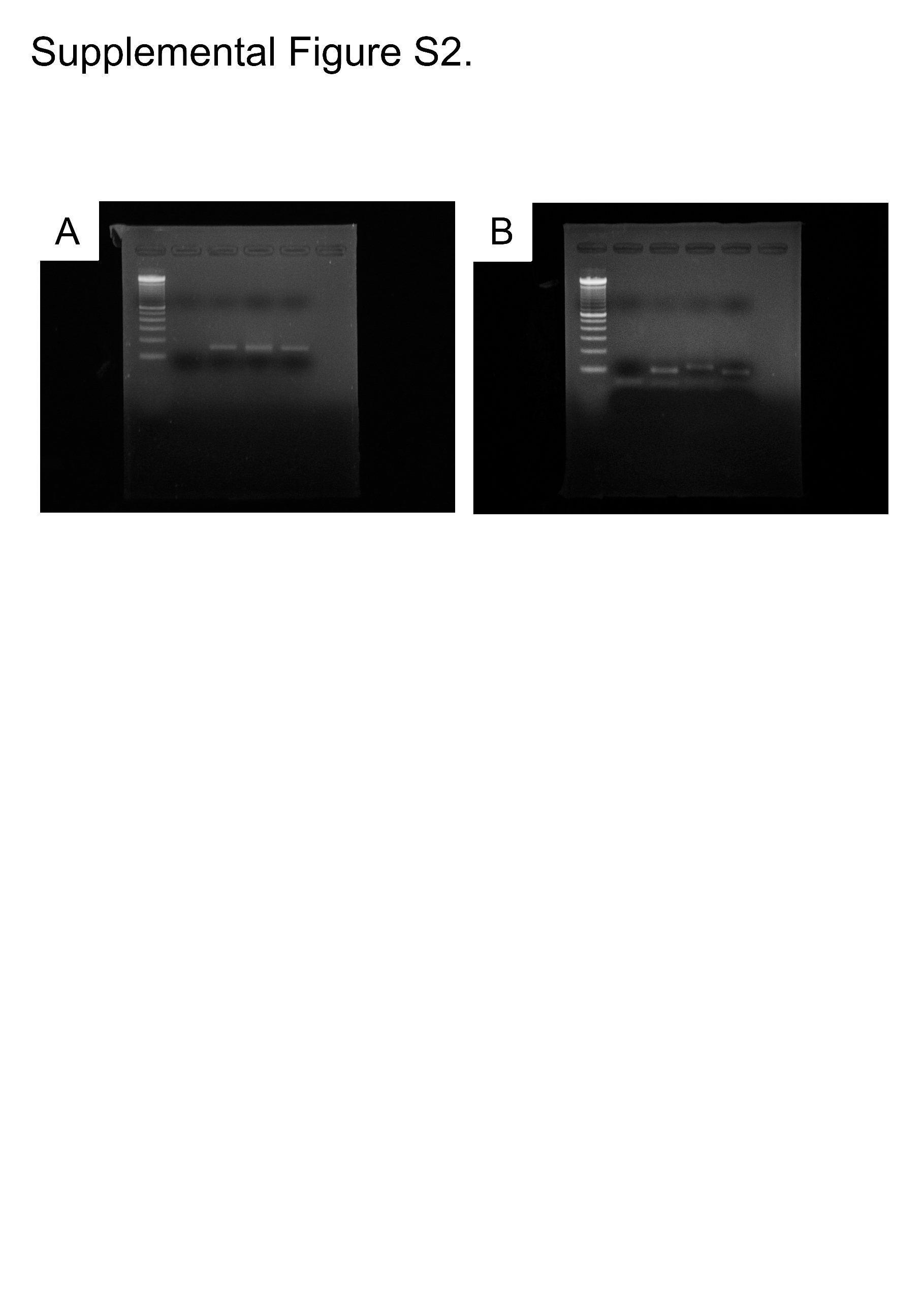


**Legends for Supplemental Figures**

**Supplemental Figure S1.**

Pathological features of pancreatic ductal carcinoma in examined subjects (H&E sections).

Tumor cell growth with irregular tubular architecture and fibrous stroma is noted in non-DM **(A)** and short-DM **(B)**. In contrast, invasive growth of tumor cells devoid of apparent tubules was pronounced in long-DM **(C)**. In non-neoplastic areas, ductal structures preserved their integrity without apparent cellular atypia in all groups **(D: non-DM, E: short-DM, F: long-DM)**. Bar represents 50μm in each.

**Supplemental Figure S2.**

Original full-length gels of methylation specific PCR for CDKN2A and CDH1.

Promoter methylation status of CDKN2A (**A**) and CDH1 (**B**) in methylation specific PCR was exhibited. Detailed explanation was described in the legend of Figure 1.

**List of supplementary material for reviewers only**

**Supplemental figure S3 for reviewers only**

Expression of oxidative stress related molecules in subjects with pancreatic ductal carcinoma (PDC) evaluated by immunohistochemistry.


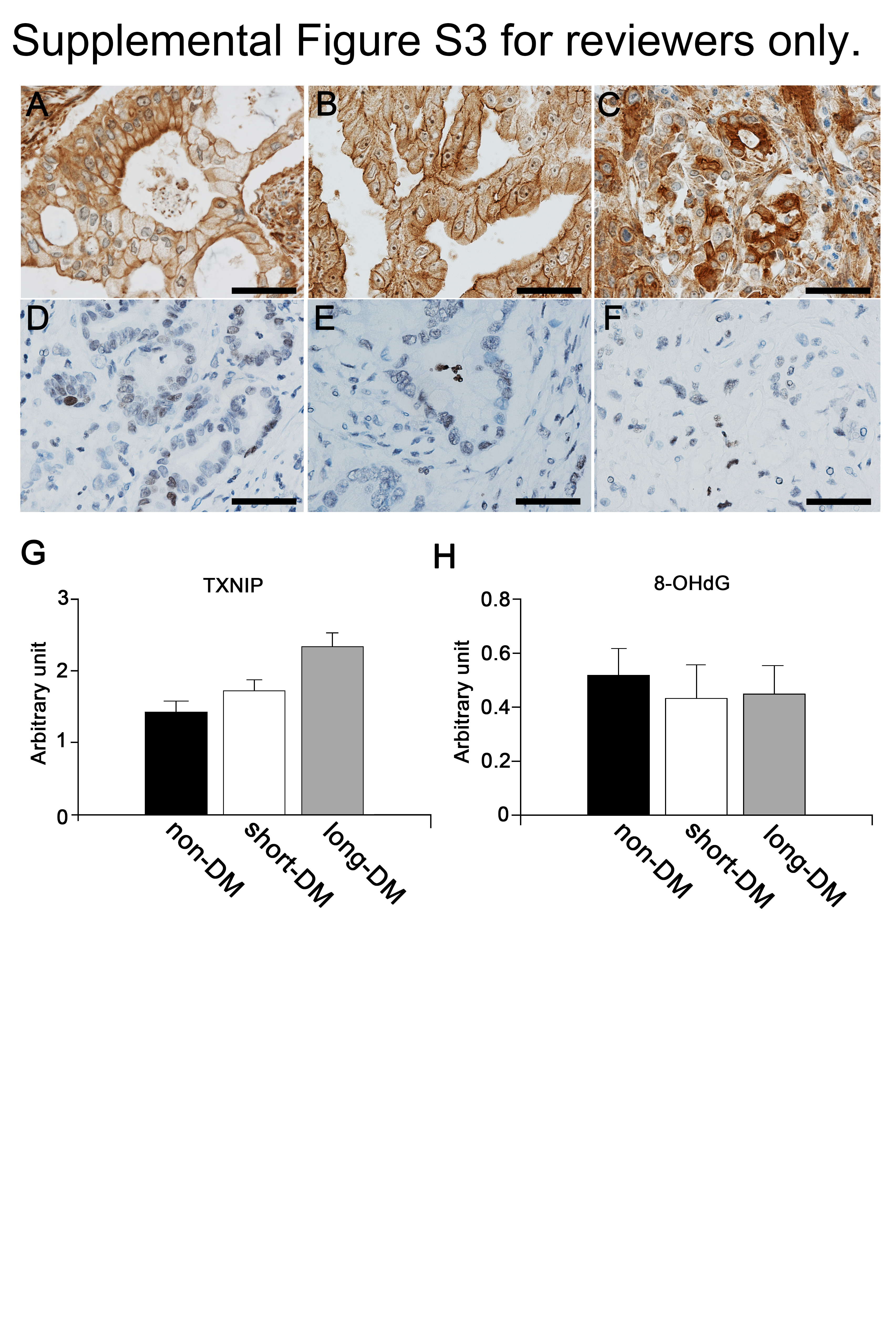


**Legends for Supplemental Figures for reviewers only.**

**Supplemental Figure S3 for reviewers only.**

Expression of oxidative stress related molecules in subjects with pancreatic ductal carcinoma (PDC) evaluated by immunohistochemistry.

Thioredoxin-interacting protein (TXNIP) was located in cytoplasm and cell membrane of tumor cells. In non-DM **(A)** and short-DM **(B)**, the expression in cytoplasm of tumor cells appeared to be low, while strong cytoplasmic expression of TXNIP in tumor cells was found in the subjects of long-DM (**C**). On the other hand, the expression of 8- hydroxy-deoxyguanosine (8-OHdG) in tumor cells was equivocal compared to stromal inflammatory cells in all groups **(D: non-DM, E: short-DM, F: long-DM)**. We preliminarily examined the cytoplasmic intensity of TXNIP expressions and nuclear intensity of 8-OHdG in tumor cells with semi-quantitative analysis which were graded into 4 grades (0; none, 1; weakly positive, 2; clearly positive, and strongly positive). In this grading, staining intensity comparable to those of normal pancreatic ductal cells were scored as 2. If the intensity was stronger than normal pancreatic ductal cells, score was 3, and lower than normal ductal cells, score was 1. As shown in the Figure, our preliminary results on a fraction of cases among the groups disclosed a trend toward an increase in the TXNIP expression in long-DM compared to in short-DM and non-DM **(G)**. There was no significant difference regarding the expression of 8-OHdG among all the groups (**H**). Bar represents 50μm in each.
